# Book Review: Drug Use and Abuse

**DOI:** 10.3389/fpsyg.2019.01817

**Published:** 2019-08-07

**Authors:** Katelyn Rinker

**Affiliations:** Department of Psychology, Washington State University, Pullman, WA, United States

**Keywords:** methylphenidate, attention, ADHD, pediatric, drug use, addiction

Stephen Maisto, Mark Galizio, Gerard J. Connors (Boston, MA: Cengage Learning), 2017, 512 pages, ISBN: 978-1337408974

Kids born in the 1990s were prone to a diagnosis of Attention-deficit/hyperactivity disorder, or ADHD (Maisto et al., 2017). This behavioral disorder presents with symptoms of excess “activity, restlessness, difficulty concentrating or sustaining attention, and impulsivity” (Maisto et al., 2017). ADHD typically relies on pharmaceutical treatments.

A psychopharmacology book written by Stephen Maisto, Mark Galizio, and Gerard Connors describes a variety of drugs in great detail. Two main groups of drugs in the book include stimulants and depressants. The drugs classified as depressants consist of alcohol and opiates, such as morphine derivatives or other analgesic drugs (Maisto et al., 2017). Yet the stimulants include nicotine, caffeine, cocaine, and the amphetamines (Maisto et al., 2017). Stimulants all have similar psychological and physiological effects on the body.

Amphetamines, methylphenidate, and other stimulants share nearly the same pharmacokinetics (Maisto et al., 2017). Most can be abused through injection into the bloodstream or intranasal administration as well as taken orally (Maisto et al., 2017). Physical effects of stimulants consist of alterations in serotonin, dopamine, and norepinephrine levels (Maisto et al., 2017). And behavioral effects include excessive personality changes, such as increases in arousal, alertness, physical endurance, and social behaviors (Maisto et al., 2017). Stimulants may also create feelings of wakefulness or energy.

But in the book's discussion on stimulants, the medication Ritalin prominently stands out. Methylphenidate carries the brand name of Ritalin, Concerta, or Daytrana. The book by Maisto and associates emphasizes the controversy of prescribing methylphenidate to children with ADHD (Maisto et al., 2017). They suggest that in 1990, use of methylphenidate has sky-rocketed at a huge increase of 500% (Maisto et al., 2017). They admit that since that date, pharmaceutical companies have earned eight billion dollars from Ritalin sales alone (Maisto et al., 2017). But this statistic was pulled from only populations of children who were old enough to attend school (Maisto et al., 2017). The *Journal of the International Neuropsychological Society* agrees that in the past 25 years, prevalence of ADHD diagnosis has increased (Mahone and Denckla, 2017).

Stephen Maisto and his team of researchers argue that any child can be diagnosed with ADHD (Maisto et al., 2017). They suggest that restlessness, fidgeting, or inattention is common among children (Maisto et al., 2017). They even go as far to say that children are labeled with ADHD due to bored teachers that do not know how to handle a child's poor performance in school (Maisto et al., 2017). This concept might spark controversy, but the book mentions some interesting points.

However, methylphenidate does improve academic performance and reduces problem-behaviors (Maisto et al., 2017). Some changes in behavior consist of the new ability to focus on a task in a timely manner (Maisto et al., 2017). As a result of this fact, methylphenidate use is commonly known as “academic doping” (Maisto et al., 2017). The *Journal of Attention Disorders* agrees that ADHD negatively impacts focus in school, which was tested on over two hundred college students (Jones et al., 2015). No matter the education level, ADHD seems to take a toll on this important area of functioning.

The effectiveness of methylphenidate is demonstrated in a research study by the *American Journal of Health System Pharmacy* (Sugrue et al., 2014). They found the medication to be a very “useful option” for pediatric patients (Sugrue et al., 2014). So it is well known that methylphenidate is a common treatment for ADHD.

But there are physical side effects of methylphenidate, such as loss of appetite and weight (Maisto et al., 2017). Insomnia can also occur when taking methylphenidate (Maisto et al., 2017). This drug is popular for keeping users awake for days on end, with little need for food or sleep.

Another treatment for ADHD are the amphetamines, such as Adderall or Vyvanse, which are closely rated to methylphenidate (Maisto et al., 2017). Amphetamines are historically used to treat the common cold, narcolepsy, obesity, and attention disorders (Maisto et al., 2017). The drug was known to cause a rush of euphoria, along with the desire to avoid sleeping or eating (Maisto et al., 2017). Later, when the 1960s began, a new era of amphetamine users were given the nickname of “speed bugs” due to their formication symptoms (Maisto et al., 2017). Formication conditions describe an individual who feels that tiny bugs are crawling under their skin, which induces pain or itching.

A drug that is shockingly similar to methylphenidate is methamphetamine, which was created in the nineteenth century (Maisto et al., 2017). This illegal drug was developed toward the end of the 1970s as a substitute for cocaine or opiates (Maisto et al., 2017). From 2004 to 2012, methamphetamine laboratories formed in the Midwest of the United States in mass numbers (Maisto et al., 2017). In 2004, California alone conjured over two thousand methamphetamine laboratories (Maisto et al., 2017). This drug was perhaps the most well-known for being created by local chemists instead of imported through other countries.

The pharmacology book authored by Stephen Maisto, Mark Galizio, and Gerard Connors dives into the controversy that methylphenidate is addictive (Maisto et al., 2017). They claim that long-term use of methylphenidate leads to a high possibility of enjoying participation in raves, which are all-night dance parties at hidden clubs (Maisto et al., 2017). Events such as these show that methylphenidate users may seek out certain activities to try to obtain more of the drug.

There is even a large market for the illegal sale of methylphenidate (Maisto et al., 2017). The drug is labeled on the street at “vitamin R” (Maisto et al., 2017). It apparently makes “users feel energetic and enhances mood,” according to the *Drug Use & Abuse* book (Maisto et al., 2017). Illegal use of methylphenidate is very popular, as over 60 percent of college students admit trying the drug (Maisto et al., 2017). So the potential for dependence on methylphenidate is extremely high. If one wishes to understand the psychopharmacology behind stimulant and depressant drugs, then Maisto's book will satisfy that desire.

## Author Contributions

The author confirms being the sole contributor of this work and has approved it for publication.

### Conflict of Interest Statement

The author declares that the research was conducted in the absence of any commercial or financial relationships that could be construed as a potential conflict of interest.
